# The Antifungal Potential of Ozonated Extra-Virgin Olive Oil Against *Candida albicans*: Mechanisms and Efficacy

**DOI:** 10.3390/biom14111472

**Published:** 2024-11-19

**Authors:** Simone Augello, Valentina Cameli, Arianna Montanari, Stefano Tacconi, Daniela Uccelletti, Luciana Dini, Emily Schifano

**Affiliations:** 1 Department of Biology and Biotechnology “Charles Darwin”, Sapienza University of Rome, Piazzale Aldo Moro 5, 00185 Rome, Italy; simone.augello@uniroma1.it (S.A.); valentina.cameli.98@gmail.com (V.C.); ari.montanari@uniroma1.it (A.M.); stefano.tacconi@uniroma1.it (S.T.); emily.schifano@uniroma1.it (E.S.); 2 Research Center for Nanotechnology Applied to Engineering, Sapienza University of Rome, Piazzale Aldo Moro 5, 00185 Rome, Italy

**Keywords:** *C. albicans*, antifungal, ozonated oil, oxidative stress, autophagy

## Abstract

The growing emergence of resistance mechanisms and side effects associated with antifungal agents highlight the need for alternative therapies. This study aims to investigate the antifungal potential of ozonated extra-virgin olive oil (EOO) against *Candida albicans*, with the goal of developing eco-friendly and highly effective treatments based on natural products. Antifungal activity was evaluated via cell viability and biofilm formation assays using Crystal Violet and Sytox green staining. The results showed that EOO reduced *C. albicans* viability in a dose-dependent manner, achieving over 90% cell death at a 3% (*v*/*v*) concentration. Transmission Electron Microscopy (TEM) revealed cell wall structural damage, and ROS levels increased by approximately 60% compared to untreated controls within 10 min of treatment. Additionally, the expression of autophagy-related genes *atg-7* and *atg-13*was upregulated by 2- and 3.5-fold, respectively, after 15 min, suggesting a stress-induced cell death response. EOO also significantly inhibited hyphal formation and biofilm development, thus reducing *C. albicans* pathogenicity while preserving cell biocompatibility. EOO antifungal activity was also observed in the case of *Candida glabrata.* In conclusion, ozonated olive oil demonstrates potent antifungal activity against *C. albicans* by reducing cell viability, inhibiting hyphal and biofilm formation, and triggering oxidative stress and autophagy pathways. These findings position EOO as a promising alternative therapy for fungal infections.

## 1. Introduction

Natural products play a crucial role in the discovery of new molecules and serve as templates for the development of synthetic drugs, ranging from anticancer agents to antimicrobials [1]. Among these, extra-virgin olive oil has gained attention for its antimicrobial properties against a broad spectrum of pathogens [2]. This superior category olive oil is obtained directly from olives (*Olea europaea*) through mechanical means, preserving many of the original bioactive components of the fruit. This unique composition, rich in antioxidants, monounsaturated fatty acids (MUFAs), and polyphenols, distinguishes extra-virgin olive oil from other vegetable oils and contributes to its health-promoting effects.

Fungal infections are a significant contributor to infectious disease-related mortality worldwide [3]. Among these, *Candida* species are the most common causes of invasive mycotic disease, with *C. albicans* being the predominant agent of invasive candidiasis. While in many healthy individuals, *C. albicans* exists as a harmless commensal in the oral cavity or gastrointestinal tract, in severely immunocompromised patients, this fungus can disseminate into the bloodstream and colonize internal organs, resulting in life-threatening systemic infections. Hyphae are recognized as one of the key structures determining the pathogenicity of this yeast. The transition from yeast-like to filamentous form is associated with increased virulence and enhanced ability to penetrate host tissues [4,5,6,7]. Due to dietary changes, antibiotic use, pH alterations, or immune system disorders, *C. albicans* can become pathogenic, rapidly multiplying and causing clinical manifestations, ranging from mucocutaneous disorders such as vaginal, oral, and intestinal candidiasis to invasive infections affecting multiple organs [8,9,10,11].

Most cases of candidiasis are linked to the formation of biofilms on biological or artificial surfaces [12]. Biofilms are complex, structured microbial communities typically encased in a self-generated matrix of exopolymeric substances [13]. During its development, *C. albicans* biofilms consistently release yeast cells with a distinctive elongated shape, which facilitates the colonization of new infection sites. This dispersal stage ensures the repetition of the “biofilm life cycle” [14]. The formation of biofilms complicates treatment and contributes to significant morbidity and cell death, making it a critical virulence factor in the pathogenesis of candidiasis [14]. Clinically, the two primary challenges posed by biofilm formation are the enhanced resistance of biofilm-embedded cells to antifungal treatments and their evasion of host immune defenses [15].

Despite the detrimental impact that fungi have on human health, only a few classes of antifungal drugs are currently available for treating these life-threatening infections. The development of novel antifungal agents has been slow, largely due to the eukaryotic nature of fungal cells, challenges associated with compound permeability across the fungal cell wall and membrane [16].

While the type of therapy varies according to the severity of the infection, common methods include the use of topical antifungals, such as antifungal creams containing agents such as clotrimazole, miconazole, or econazole, and oral antifungals containing fluconazole [17,18]. However, in recent decades, antifungal agents have become increasingly ineffective due to the emergence of resistance mechanisms such as target alteration and overexpression. Furthermore, the limited number of existing antifungal classes raises concerns [3,19,20]. In addition to resistance problems, antifungal treatment may interact with other drugs a person is taking, affecting the effectiveness of both and increasing the risk of side effects [21]. Prolonged use of antifungals can also lead to dysbiosis, a condition in which the bacterial flora is altered [22]. Therefore, there is a growing need for the development of alternative therapies, focusing on new antifungal targets and the use of active compounds from different sources, including natural products [23,24].

Among these, ozonated oils are gaining interest due to their multiple applications and antimicrobial activity [25]. Ozone (O_3_) is a highly reactive molecule consisting of three oxygen atoms, functioning as both an oxidant and an oxidizer [26]. O_3_ exposure induces oxidative stress by promoting amino acid oxidation, cell wall reactions, and DNA damage, ultimately leading to cell death [27]. Oxidative stress primarily results from the production of intracellular reactive oxygen species (ROS), which are closely linked to both normal physiological homeostasis and disease pathogenesis. Cellular ROS levels and oxidized components can be mitigated by autophagy, a self-clearance pathway [28]. ROS can modulate autophagy activity through transcriptional and post-translational mechanisms. Autophagy, in turn, activates transcription factors and degrades damaged organelles and proteins, reducing excessive ROS within cells. In this way, autophagy serves an antioxidant role, protecting cells from oxidative stress. However, excessive autophagy can lead to autophagic cell death.

The aim of this study is to investigate the effects of ozonated extra-virgin olive oil using various ozonated oil concentrations and different numbers of ozonides on planktonic cell growth, biofilm formation, and hyphal growth of *C. albicans*, elucidating the mechanism of action through gene expression analysis, TEM imaging, and reactive oxygen species (ROS) production assays.

## 2. Materials and Methods

### 2.1. Olive Oil Ozonation and Quantitative Determination of Ozonides

A commercial extra-virgin olive oil was used for the ozonation process, carried out by bubbling ozone gas with the OZONIS STERIL 250 series ozonator. This ozonator is powered by air from which ozone is produced at a constant gas flow rate of 1.5 L/min allowing a production of 250 mg/h of ozone. To produce ozonated oil, 20 mL of olive oil was ozonated at 25 °C for different times of ozonation (4, 8, and 24 h).

The analysis of ozonide content was carried out by titration with sodium thiosulfate using a 50 mL burette. Then, 0.3 g of ozonated oil was placed in a 100 mL conical flask and mixed with 3 mL of chloroform (CHCl_3_) and 4.5 mL of acetic acid (CH_3_COOH) under agitation. After adding 350 µL of saturated potassium iodide solution, 5 mL of H_2_O_dd_ was added. The final solution was titrated with a 0.01 N sodium thiosulphate solution (0.08 g of Na_2_S_2_O_3_ in 100 mL of H_2_O_dd_) until the color changed. The number of ozonides was calculated with the following formula:

$$\frac{Volume\;of\;sodium\;thiosulfate\;used\;\times \;normality\;of\;sodium\;thiosulfate\;\left(0.01\;N\right)\;\times \;1000}{0.3\;g\;of\;ozonated\;oil\;used}$$

### 2.2. Triglyceride Composition of Ozonated Olive Oil

Triglycerides present in the oil were subjected to a transesterification process with methanol (CH_3_OH) in the presence of an acid catalyst (BF_3_), releasing glycerol and the methyl esters that constitute the triglycerides. In a vial, 10 µL of oil was added to 2 mL of benzene and 1 mL of CH_3_OH/BF_3_ solution. The vial was then immersed in a hot water bath at 90 °C for 20 min. Afterward, the vial was cooled and 2 mL of H_2_O_dd_ was added, followed by vigorous shaking. After a few seconds, two distinct layers were observed: an aqueous phase containing glycerol and an organic phase containing the methyl esters of fatty acids. The organic phase was analyzed by injecting an aliquot of the sample into a gas chromatograph and gas chromatograph–mass spectrometer (GC-MS). The mass spectrometer was operated in EI mode at 70 eV using Agilent MSD ChemStation D.01.00 software. The peaks in the gas chromatogram were identified and assigned to various fatty acids based on their retention times and molecular weights. This allowed for qualitative analysis and the determination of the percentage composition of each fatty acid by calculating the area of each peak.

### 2.3. Cell Viability Assay

*Candida albicans* ATCC 10231 and *C. glabrata* ATCC 2001 (CBS 138) were cultured in Yeast Peptone Dextrose (YPD) at 28 °C under aerobic conditions. The viability of *Candida cells* following treatment with ozonated extra-virgin olive oil was assessed using the Colony Forming Unit (CFU) assay. An aliquot of 1 × 10^8^ cells/mL from an overnight culture was incubated in YPD at 28 °C with shaking in the presence of different concentrations of ozonated olive oil (1%, 2%, or 3% (*v*/*v*)) and different number of ozonides (386, 853, or 2492). At the indicated time points (1, 2, or 4 and 24 h), samples were diluted and plated onto YPD agar plates. Yeast cultures grown under identical conditions but without the addition of ozonated oil served as controls. The survival rate was calculated using the following equation: [(CFU/mL) of the treated cells/(CFU/mL) of the untreated sample] × 100.

### 2.4. Ergosterol Binding Assay

An overnight culture of *C. albicans* (1 × 10^8^ cell/mL) was centrifuged, and the resulting pellet was washed with Phosphate Buffered Saline (PBS). The pellet was then resuspended in PBS containing different concentrations of exogenous ergosterol (ranging from 50 to 200 µg/mL) and 3.0% (*v*/*v*) ozonated oil (853 ozonides). After 2 h of incubation at 28 °C under aerobic conditions, the ability of *C. albicans* to form colonies was assessed using the CFU counting method.

### 2.5. Sorbitol Effect Assay

An aliquot containing 1 × 10^8^ cells/mL of *C. albicans* overnight culture was centrifuged and washed once with PBS, and the resulting pellet was resuspended in 2 mL of PBS supplemented with sorbitol (0.8 M), an osmoprotectant for fungal cell wall, and 3.0% (*v*/*v*) ozonated oil (853 ozonides). Following incubation at 28 °C with agitation for the indicated timing, the capability of *C. albicans* to form colonies was assessed using the CFU method.

### 2.6. Evaluation of Intracellular ROS Levels

Approximately 5 × 10^7^ cells/mL derived from an overnight culture of *C. albicans* were incubated in YPD medium supplemented with a concentration of 3% (*v*/*v*) ozonated oil (853 ozonides) and increasing concentrations of ascorbic acid (50–100–500 µg/mL) for 10, 30, and 60 min at 28 °C with shaking. Subsequently, following a wash with PBS, staining for the detection of reactive oxygen species (ROS) was performed using 20 µM dihydroethidium (DHE). The samples were then incubated at 37 °C for 15 min in the absence of light. After another wash with PBS, the fluorescence was detected by using the Zeiss Axiovert 25 inverted fluorescence microscope. The detection of ROS was assessed by evaluating the number of positive cells for ROS production compared to the total number of counted cells.

### 2.7. Hyphal Growth

An aliquot of 1 × 10^7^ cells/mL of a *C. albicans* overnight culture were inoculated in YPD medium supplemented with 10% Fetal Bovine Serum (FBS) along with ozonated oil (853 ozonides). Following incubation at 37 °C for 48 h, filamentous cells were examined under a microscope using the fluorescent dye calcofluor white (CFW). The cells were washed twice with PBS, and then, 10 mg/mL CFW was added for 10 min in the dark at 25 °C. Finally, hyphal growth was observed under a Zeiss Axiovert 25 fluorescence microscope using a UV filter.

For the induction of hyphal growth on solid media, *C. albicans* was spotted onto Spider agar medium containing 3.0% (*v*/*v*) ozonated oil (853 ozonides). After 5 days of incubation at 37 °C, hyphal growth was observed using a stereoscope Nikon SMZ25.

### 2.8. Biofilm Evaluation Using Crystal Violet Quantitative Assay

A *C. albicans* biofilm was induced by inoculating a single colony in Yeast Nitrogen Base (YNB) supplemented with 100 mM glucose under agitation overnight at 28 °C. Subsequently, the cells were washed twice in PBS and resuspended at a concentration of 1 × 10^7^ cells/mL in PBS. A total of 500 µL of the resuspended cells was transferred to each well of a 24-well flat bottom microtiter plate and incubated at 37 °C for 1.5 h under static conditions to promote the biofilm adhesion phase. After this phase, non-adherent cells were removed by washing with PBS, and 1 mL of YNB medium supplemented with different concentrations of ozonated oil (853 ozonides), ranging from 0.5% to 4.0% (*v*/*v*), was added to each well. The plate was then incubated under anaerobic conditions at 37 °C for 48 h. Following incubation, the medium was removed, and two washes with PBS were performed. Then, 1 mL of 0.3% Crystal Violet dye was added to each well, and after incubating for 15 min in the dark, the dye was removed. Finally, 1 mL of 96% ethanol was added to each well, and after dissolving the dye, the absorbance was measured at OD 600 nm using a spectrophotometer.

### 2.9. Sytox-Green Staining

An aliquot of 1 × 10^6^ cells/mL derived from an overnight culture of *C. albicans* was inoculated in Spider medium with 0.5%, 1.5%, 2.5%, or 3.0% (*v*/*v*) ozonated oil (853 ozonides) in 35 mm plates containing a sterile coverslip (24 × 24 mm). After incubation at 37 °C for 48 h, the samples were gently washed with PBS, and 1 μM SYTOX dye was added onto the coverslip. After 15 min, a final wash with PBS was performed, and the coverslip was mounted and observed under a Zeiss Axiovert 25 fluorescence microscope (Jena, Germany) using a FITC filter.

### 2.10. TEM Observation

A colony of *C. albicans* was inoculated into 10 mL of YPD medium and incubated with shaking overnight at 28 °C. A cell suspension of 1 × 10^8^ cells/mL was supplemented with ozonated oil (853 ozonides) at a final concentration of 3.0% (*v*/*v*).

Following 30 min treatment, the cells were transferred to 2 mL Eppendorf tubes and washed with sterile PBS through centrifugation at 3500× *g* for 1 min. Subsequently, the pellet was fixed and embedded as reported in [29]. Thin sections of 60 nm were cut at the ultramicrotome and deposited on 200 mesh formvar/carbon-coated grids. All observations were performed with transmission electron microscope JEOL 1400JEM (Tokyo, Japan) operating at 80 kV equipped with a camera Orius 600 gatan and Digital Micrograph (Product version: 1.7) [30].

### 2.11. Real Time qPCR

RNA of 10^8^ *C. albicans* cells was extracted using miRNeasy Micro Kit (Qiagen) and real time analysis was carried out with I Cycler IQ Multicolor Real-Time Detection System (Bio-Rad Laboratories, Hercules, CA). Quantification was performed using a comparative *C*_T_ method (*C*_T_ = threshold cycle value). Briefly, the differences between the mean *C*_T_ value of each sample and the *C*_T_ value of the housekeeping gene (*act-1*) were calculated: Δ*C* _Tsample_ = *C* _Tsample_—*C* _A*CT1*_. Result was determined as 2^−ΔΔCT^ where ΔΔ*C* _T_ = Δ*C* _Tsample_ − Δ*C* _Tcontrol_. The experiment was performed in triplicate.

### 2.12. Biocompatibility Evaluation

The biocompatibility of ozonated oil was tested on human epidermal keratinocyte HaCaT cells, spontaneously immortalized from a primary culture of keratinocytes. The cells were seeded at a density of 4 × 10^3^ cells/well in 96-well plates in DMEM (Aurogene, Rome, Italy), supplemented with 10% FBS, 2 mM L-Glutamine, and 100 units/mL penicillin and 100 mg/mL streptomycin (Sigma-Aldrich, St. Louis, MO, USA) at 37 °C with 5% CO_2_ in a humidified atmosphere. After 24 h, the cells were treated with 1% or 3% of ozonated oil in complete DMEM medium for 1 h and 3 h. Untreated cells were used as a control. After the treatments, cell viability was assessed as reported in [31].

## 3. Results

### 3.1. Ozonated Extra-Virgin Olive Oil Exerted Antifungal Activity on Candida albicans Cultures

**Figure 1:**
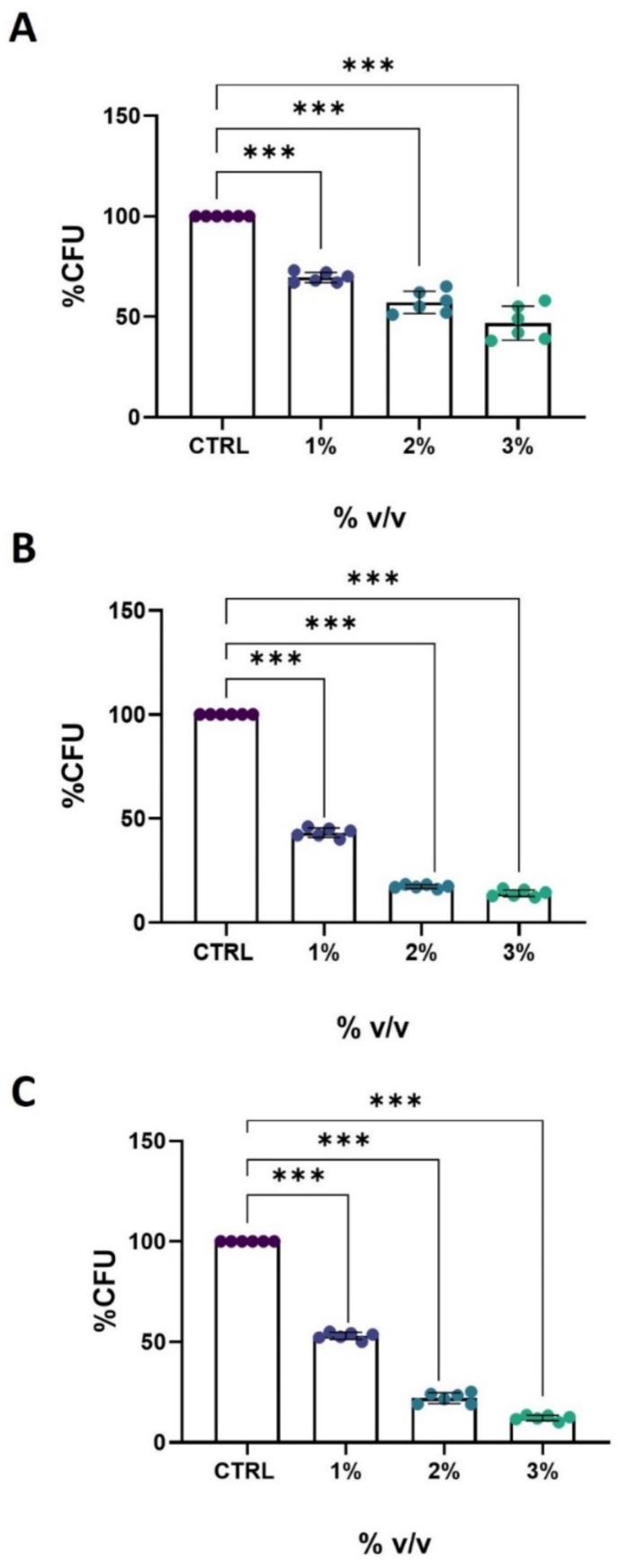
Evaluation of ozonated oil on *C. albicans* viability through Colony Forming Unit (CFU) method. (**A**) Effect of ozonated oil 386 ozonides, (**B**) 853 ozonides, or (**C**) 2492 ozonides using different oil concentrations as indicated. Untreated cells (CTRL) were taken as a control. Experiments were performed in triplicate. Data are presented as mean ± SD, and one-way ANOVA analysis with the Bonferroni post test was used (*** *p* < 0.001 with respect to CTRL).

The antifungal activity of extra-virgin ozonated olive oil (386, 853, or 2492 ozonides) was evaluated by assessing the viability of *C. albicans* cells following 24 h of treatment with different oil concentrations (1%, 2%, or 3% (*v*/*v*)) (Figure 1). At a concentration of 1% (*v*/*v*), the 386 ozonides oil exhibited noticeable antifungal efficacy, resulting in a 35% reduction compared to the untreated control. The efficacy increased with concentration, with a viability reduction of 50% observed at a concentration of 3% (*v*/*v*) (Figure 1A). Notably, the 853 or 2492 ozonide oils demonstrated greater effectiveness, achieving a reduction in yeast growth of approximately 50% even at the lowest concentration of 1% (*v*/*v*). Furthermore, at the highest concentration of 3% (*v*/*v*), these oils resulted in more than 90% cell death (Figure 1B,C).

**Figure 2:**
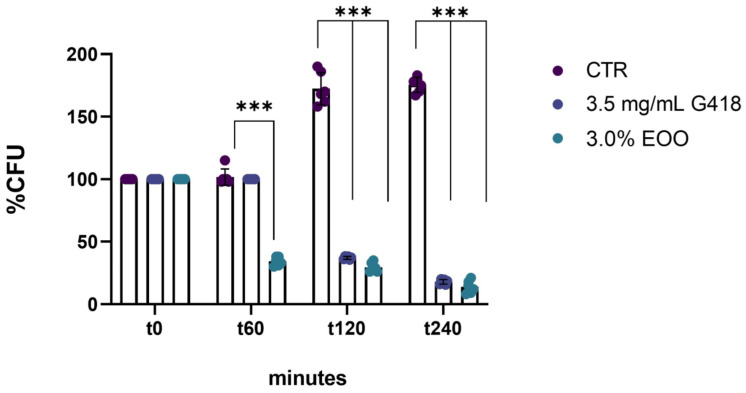
Effect of ozonated oil on *C. albicans* at different time points. The percentage of cells recovered after treatment for 0, 1 h, 2 h, and 4 h with ozonated oil (853 ozonides) at a concentration of 3.0%. Untreated cells (CTR) were taken as a control, and 3.5 mg/mL G418 was used as the antifungal standard. Experiments were performed in triplicate. Data are presented as mean ± SD (*** *p* < 0.001;).

Following the assessment of cell viability with 24 h ozonated olive oil treatment, the efficacy of shorter exposure times was evaluated. Treatments for 1, 2, and 4 h with 853 ozonated oil at a concentration of 3% (*v*/*v*) were examined. One hour after treatment, the cell viability of *C. albicans* already decreased by 65% compared to the untreated control. Additionally, it was observed that the antifungal effect of ozonated olive oil is dependent on exposure time; notably, after 4 h of treatment, cell viability was significantly reduced by 95% compared to untreated cells (Figure 2).

### 3.2. Alterations in Triglyceride Composition of Oil Following Ozonation Process

The data presented in Table 1 demonstrate the changes in the triglyceride composition of the oil depending on the ozonation process. Significant decreases in the content of the oleic acid and linoleic acid were observed in the oil after ozonation with respect to the olive oil. Three compounds were formed after the ozone treatment. While palmitic acid remained almost in the same concentration, the gas chromatography analysis revealed the presence of nonanal, nonanoic acid, and trans oleic acid only in the ozonated olive oil. These compounds are the results from the reaction of ozone with the double bond in the fatty acid that led also to an increase in the stearic acid.

### 3.3. Ozonated Olive Oil Impacts on Cell Wall Biosynthesis

**Figure 3:**
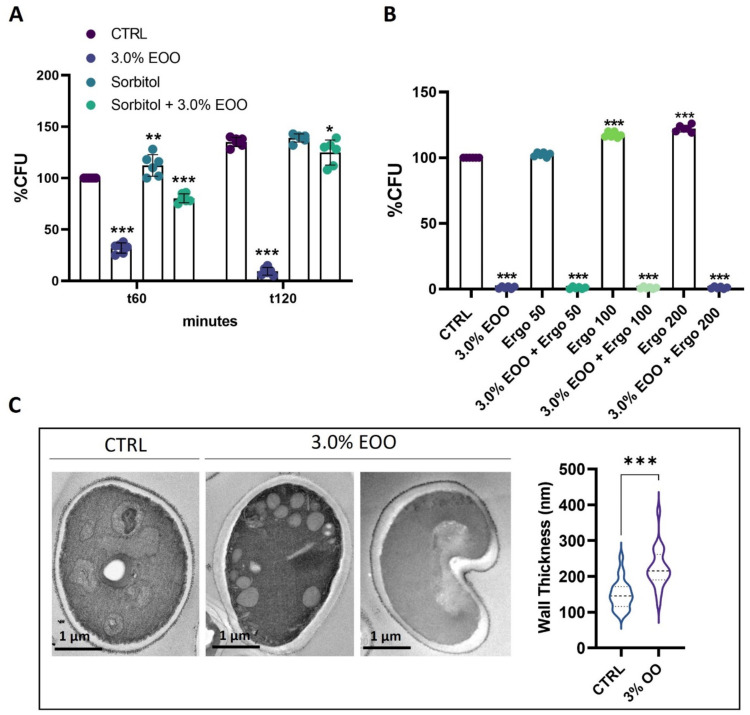
Analysis of ozonated oil on *C. albicans* cell wall. (**A**) The experiment was performed at different time points of treatment using extra-virgin ozonated olive oil (EOO) with 853 ozonides. Untreated (CTR), treated only with ozonated oil (EOO), treated only with sorbitol, and treated with sorbitol and EOO. (**B**) Cells were treated for 2 h with ozonated oil (853 ozonides) at 3% (*v*/*v*), and different concentrations of ergosterol: 50; 100; and 200 µg/mL (Erg). Untreated cells (CTRL) were taken as a control. (**C**) Ultrastructural analysis of the *C. albicans* treated with 3% (*v*/*v*) ozonated olive oil with 853 ozonides and wall thickness measurements. Untreated cells (CTRL) were taken as a control. Experiments were performed in triplicate. Data are presented as mean ± SD (* *p* < 0.05, ** *p* < 0.01, and *** *p* < 0.001;). Effect of ozonated oil on *C. albicans* at different time points.

To explore the antifungal mechanism underlying the action of ozonated olive oil against *C. albicans*, two compounds known to target distinct cellular components, sorbitol and ergosterol, were employed (Figure 3). The findings revealed that treatment with sorbitol, an osmotic protectant, in combination with 3.0% (*v*/*v*) ozonated oil (853 ozonides), led to an increase in cell viability compared to treatment with ozonated oil alone, already after 1 h of treatment (Figure 3A). This suggests that the antifungal activity of ozonated olive oil disrupts the functionality of the cell wall. Conversely, treatment with different concentrations of ergosterol, a component of the fungal cell membrane, in the presence of 3.0% (*v*/*v*) ozonated oil (853 ozonides) did not yield positive effects on cell viability, producing outcomes like those observed with ozonated oil treatment alone (Figure 3B). The above data suggested that distinctive alterations in cell wall organization were present in the treated cells; we then investigated the morphological aspects of these changes by electron microscopy of conventional ultra-thin sections. The analysis was performed on cells treated with 3% (*v*/*v*) ozonated olive oil for 30 min, and fixed and stained with potassium permanganate. The most evident morphological difference among the treated and untreated cells was the thickness and the architecture of the cell wall (Figure 3C). Untreated cells showed a normal structure of the cell wall (Figure 3C upper panel) all around the entire body of the cell, with the dark-stained outer layer composed of mannoproteins and the amorphous layer composed of 1,3-β- and 1,6-β-D-glucans. The treated cells showed changes in thickness and organization of the cell wall (Figure 3C middle panel). The thickness of the cell wall of the ozonized cells increased with respect to that of the control counterpart. The dark-stained mannoprotein layer was evident and less well defined in the treated ones, and the rough aspect of the external surface was characterized by a reduced electron-dense material. Moreover, an invagination of the cells was observed for 30% of the treated cells already after 30 min of incubation with ozonated olive oil, suggesting collapse of the cells (Figure 3C lower panel).

### 3.4. Ozonated Olive Oil Enhanced Oxidative Stress in C. albicans

**Figure 4:**
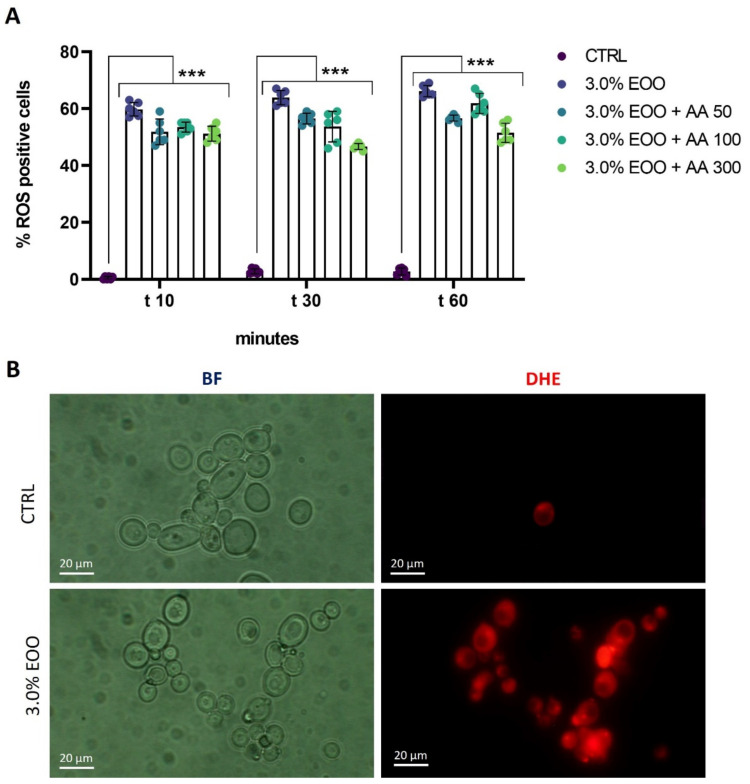
Impact of ozonated oil treatment on oxidative stress (**A**,**B**). Evaluation of ROS levels after treatment with ozonated olive oil. *C. albicans* was treated with extra-virgin ozonated olive oil (EOO) with 853 ozonides and different concentrations of ascorbic acid: 50; 100; and 500 µg/mL (AA) for different time points, as indicated. Untreated cells (CTRL) were taken as a control. Experiments were performed in triplicate. Data are presented as mean ± SD (*** *p* < 0.001 with respect to CTRL).

To investigate early intracellular damage induced by ozonated oil, the potential induction of oxidative stress was analyzed. This was achieved by staining with dihydroetidium (DHE), a probe capable of detecting reactive oxygen species (ROS), particularly superoxide anions [27]. The levels of ROS in untreated cells and cells treated with ozonated olive oil (853 ozonides) at a concentration of 3.0% (*v*/*v*) were measured after 10, 30, and 60 min of treatment (Figure 4). The results were quantified as the percentage of cells exhibiting a positive DHE signal relative to the total cell population. As reported in Figure 4A, the ozonated olive oil induced an increase in ROS in *C. albicans* already after 10 min of treatment. The inclusion of various concentrations of ascorbic acid (AA), a well-established antioxidant that neutralizes multiple types of reactive oxygen species (ROS), did not attenuate the ROS levels induced by ozonated olive oil (Figure 4A). Fluorescence images of DHE-stained cells are reported in Figure 4B.

### 3.5. Ozonated Olive Oil Induces Autophagy in C. albicans via Upregulation of atg7 and atg13

**Figure 5:**
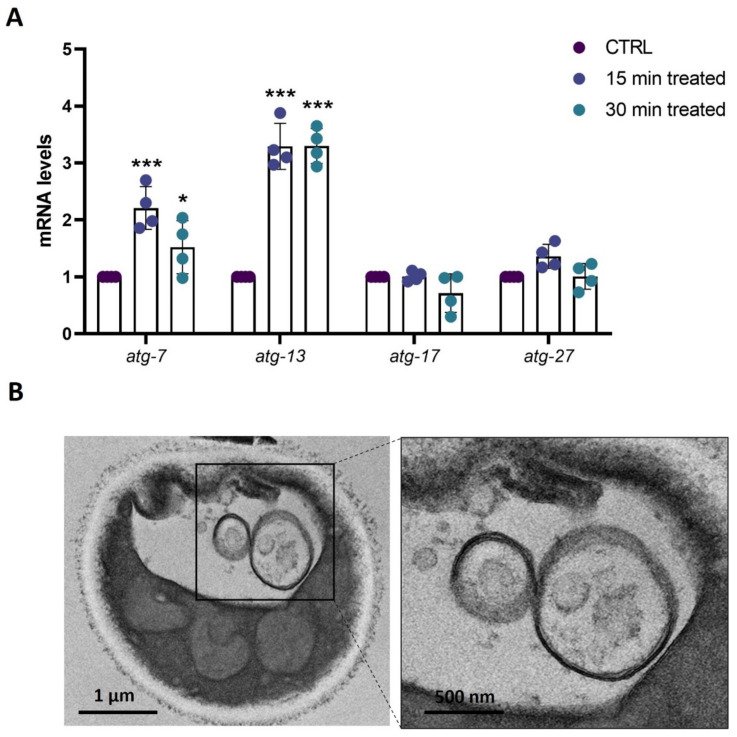
Evaluation of autophagy induction in *C. albicans*-treated cells. (**A**) Histograms show the expression of genes involved in the autophagy process detected by real-time PCR in cells treated with 3% (*v*/*v*) ozonated olive oil with 853 ozonides for different time points, as indicated. Untreated cells (CTRL) were taken as a control. Experiments were performed in triplicate. Data are presented as mean ± SD (* *p* < 0.05and *** *p* < 0.001). (**B**) Ultrastructural analysis of the *C. albicans* treated with 3% (*v*/*v*) ozonated olive oil with 853 ozonides, highlighting the presence of autophagosome.

Autophagy is a process essential for cellular homeostasis, regulating the balance between organelle biogenesis, protein synthesis, and the clearance of cells. It occurs under several conditions such as oxidative stress and has indeed emerged as a critical mediator of pathological responses associated with ROS in both cellular signaling and damage [28]. Possible activation of this process was thus investigated in the early treatment of yeast cells with ozonated olive oil. The RT-qPCR results showed changes in the autophagy level of *C. albicans*-treated cells, where four critical autophagy-related genes were selected (Figure 5A). RT-qPCR revealed that the gene expressions of *agt7* and *agt13* were significantly upregulated already after 15 min of treatment with ozonated oil compared with the control group, while the expressions of *atg17* and *atg27*were unchanged with the treatment (Figure 5A). Since autophagosomes can be detected when autophagy occurs, a transmission electron microscope analysis has been performed. Representative TEM images revealed a typical eukaryotic cell morphology, whereas *C. albicans* cells treated for 30 min with ozonated olive oil displayed a higher number of autophagosomes with well-defined membranes and distinct edges (Figure 5B). These observations indicate that the treatment with ozonated olive oil enhances the formation and visibility of autophagosomes in *C. albicans*.

### 3.6. Ozonated Olive Oil Suppresses Hyphal Formation in C. albicans

**Figure 6:**
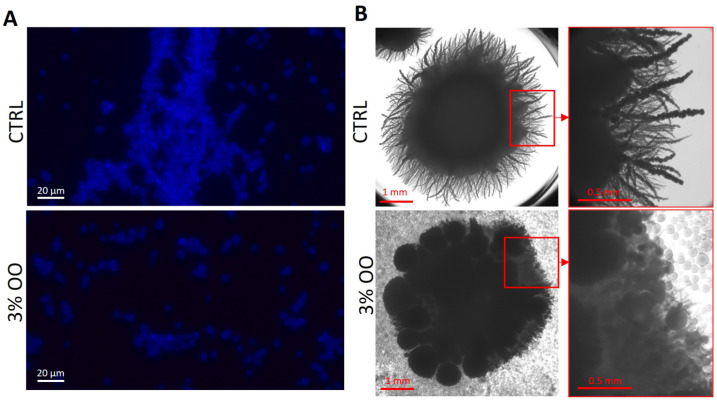
*C. albicans* hyphal growth. The treatment was performed using 3% (*v*/*v*) ozonated olive oil with 853 ozonides. (**A**) After treatment, the hyphal growth was stained with calcofluor white and evaluated by fluorescence microscopy. (**B**) Stereoscope images of *C. albicans* untreated and treated cells with 3% (*v*/*v*) ozonated olive oil with 853 ozonides, respectively.

Among the virulence factors that have a role in fungal infections, the most significant characteristic is the morphological yeast-to-hyphal transition. In fact, it induces a rapid response to different environments, as well as during host infection [29]. To assess the impact of ozonated oil on the growth of the filamentous form of *C. albicans*, ozonated olive oil (853 ozonides) was examined at 3.0% (*v*/*v*) concentrations in liquid medium, using CFW staining during a 48 h treatment period (Figure 6A). Indeed, the dye can bind chitin along the cell wall. The treatment induced an almost complete suppression of hyphal formation with respect to the untreated counterpart, suggesting a reduction in the pathogenicity. To further support this data, an analysis of hyphal formation in solid medium was also performed; *C. albicans* cells were grown in yeast form in liquid medium and then plated onto solid Spider medium. The appearance of colonies after 5 days of growth at 37 °C can be seen in Figure 6B. As expected, the untreated *C. albicans* strain formed colonies with long hyphae; in contrast, the presence of ozonated olive oil induced a clear lack of filamentation.

### 3.7. Extra-Virgin Ozonated Olive Oil Exerted Antibiofilm Effect

**Figure 7:**
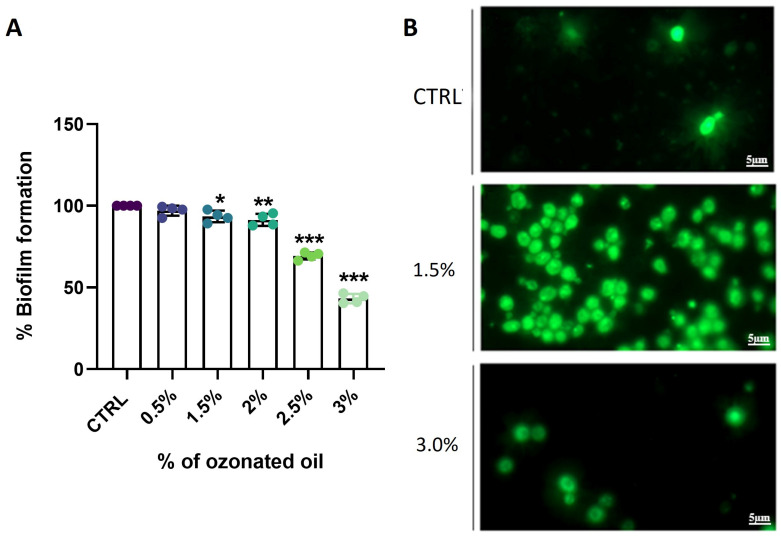
Effect of ozonated olive oil on *C. albicans* biofilm formation. (**A**) The biofilm of *C. albicans* was formed in the presence of different concentrations of ozonated oil with 853 ozonides, as indicated, and evaluated using a Crystal Violet assay. (**B**) The cells of *C. albicans* were treated with different concentrations of ozonated olive oil, as indicated, and stained with Sytox green. Untreated cells were indicated as CTRL. Data are presented as mean ± SD (* *p* < 0.05, ** *p* < 0.01, and *** *p* < 0.001).

Since *C. albicans* primarily exists in the form of biofilms during infections. Therefore, the antimicrobial activity of ozonated olive oil was assessed on biofilm formation using the Crystal Violet and the fluorescent dye SYTOX Green, known for its ability to penetrate cells with compromised cell integrity [30]. Following the treatment with ozonated oil containing 853 ozonides at varying concentrations (0.5%, 1.5%, 2.5%, or 3.0% (*v*/*v*)), biofilm reduction was observed through Crystal Violet staining (Figure 7A). Specifically, the results demonstrated a significant dose-dependent decrease in biofilm formation compared to the untreated control. A parallel analysis with SYTOX Green confirmed the reduction in biofilm compared to the untreated cells (Figure 7B). The concentrations of 1.5% and 3% (*v*/*v*) showed that most of the cells died, highlighted by a high number of SYTOX-positive cells observed, indicating compromised cell membranes.

### 3.8. Antifungal Activity Against Candida glabrata Pathogen

**Figure 8:**
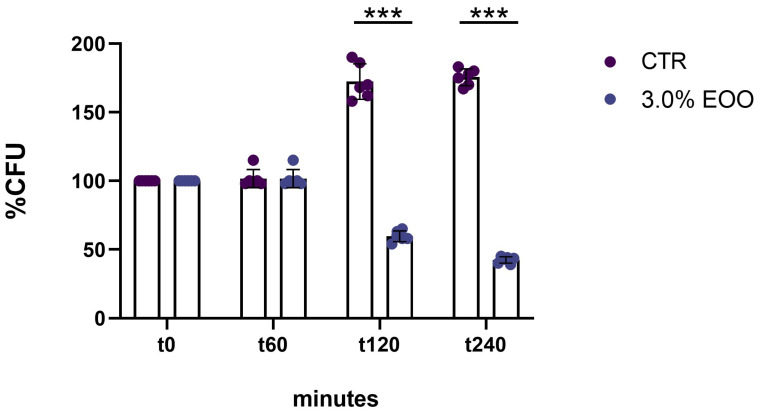
Effect of ozonated oil on *C. glabrata* at different time points. The percentage of cells recovered after treatment for 0, 1 h, 2 h, and 4 h with ozonated oil (853 ozonides) at concentration of 3.0%. Untreated cells (CTR) were taken as a control. Experiments were performed in triplicate. Data are presented as mean ± SD (*** *p* < 0.001).

Whereas *C. albicans* has been extensively studied, the antifungal activity of other non-*Candida albicans* species, such as *C. glabrata*, remains less understood. *C. glabrata* is an emerging opportunistic pathogen associated with mucosal infections, tissue invasion, and severe systemic infections, sometimes leading to mortality. Unlike *C. albicans*, *C. glabrata* is a haploid and asexual yeast that does not form hyphae, reducing its invasive capabilities but often increasing its resilience against standard antifungal treatments [32]. In this study, the efficacy of ozonated oil was also evaluated against *C. glabrata*. Treatments for 1, 2, and 4 h with 3% (*v*/*v*) ozonated oil containing 853 ozonides demonstrated potent antifungal activity. The 1 h treatment maintained cell viability comparable to the untreated control, while 2 h of exposure significantly reduced the *C. glabrata* cell viability of about 40% of cells compared to the untreated cells. After 4 h, a marked reduction of 60% in cell viability was observed (Figure 8).

### 3.9. Biocompatibility of EOO with Human Skin Cell Line

**Figure 9:**
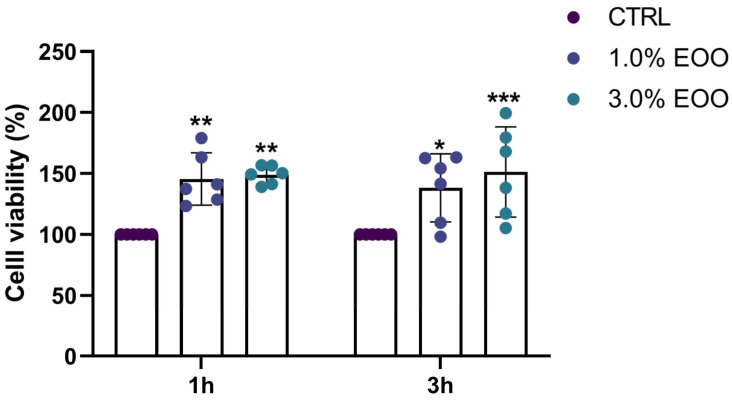
Biocompatibility assessment of EOO on the HaCaT human cell line. Cell viability was evaluated at 1 h and 3 h of treatment with 1% (*v*/*v*) or 3% (*v*/*v*) EOO. Untreated cells were used as a control (CTRL), and data were expressed as a percentage with respect to the untreated cells. Data are presented as mean ± SD, n = 6 (* *p* < 0.05; ** *p* < 0.01; *** *p* < 0.001).

To evaluate cytotoxicity, two concentrations of EOO were tested on HaCaT human skin keratinocytes (Figure 9) and compared to the untreated cells. The results showed that the EOO not only had significantly no cytotoxic effect compared to the control, but it was able to increase the vitality of treated cells in comparison to the untreated ones (150% versus 100%).

## 4. Discussion

*C. albicans*, a commensal yeast in the normal microbiota of healthy humans, is associated with a high mortality rate due to its expression of multiple virulence factors that facilitate the invasion of host tissues leading to various infections, particularly when the host’s immune system is compromised [33,34]. During the initial stage of *Candida* biofilm development, individual cells adhere to biotic or abiotic surfaces, forming a complex network of hyphae and pseudohyphae, developing into a mature biofilm enveloped in an extracellular matrix [12]. Biofilms promote the spread of fungal cells and protect them from the host immune system, reducing their susceptibility to many antifungal treatments [35]. In hospitalized patients, most fungal infections are closely linked to biofilm formation on medical devices, which directly correlates with increased mortality. Consequently, biofilms pose a significant threat to human health due to their resistance to antifungal drugs. Current antifungal treatments are insufficient in eradicating biofilm-associated infections, and the rise of multi-resistant fungal strains has become a major health concern in recent years [36].

This study demonstrated that ozonated extra-virgin olive oil exhibits significant antifungal activity against *C. albicans*, affecting both planktonic cells and biofilm formation. Especially with higher concentrations of ozonides (853 and 2492 ozonides), it significantly reduced the viability of *C. albicans* cells. At the highest concentration (3% (*v*/*v*)), ozonated olive oil containing 853 ozonides caused the death of more than 90% of the yeast cells within just 4 h of treatment, suggesting that it can effectively inhibit *C. albicans* growth and potentially offer a new avenue for antifungal therapy. One of the critical virulence factors of *C. albicans* is its ability to transition from yeast to hyphal form. The significant reduction in hyphal formation in the presence of ozonated olive oil underscores its potential to mitigate *C. albicans* pathogenicity. Both liquid and solid medium assays confirmed this suppression, suggesting that ozonated olive oil could prevent the morphological changes necessary for invasive infection. The antifungal efficacy varied with the concentration of ozonides and the exposure time, demonstrating a clear dose–response relationship. This study introduces a novel approach by evaluating the combined effects of varying concentrations of ozonated olive oil and different numbers of ozonides on yeast cells. Moreover, while different studies have explored the antifungal properties of ozonated oils on vegetative cells [37,38,39], this research uniquely addresses the impact of these variables on hyphal morphology, highlighting a critical aspect of *C. albicans* virulence that has not been previously investigated in such detail.

Ozone, a chemical compound consisting of three oxygen atoms, has a higher energy content than atmospheric oxygen and finds applications in medicine, industry, and agriculture [40]. Ozonated vegetable oils, which trap ozone in unsaturated fatty acids as ozonides, act as reservoirs that allow a slow release of ozone over time [39]. Ozonated extra virgin olive oil combines the antimicrobial properties of ozone with the beneficial properties of extra virgin olive oil due to its high content of monounsaturated fatty acids and polyphenols, making it an effective and non-invasive natural product in ozone therapy [41,42,43,44,45].

Although ozonated extra-virgin olive oil is still relatively underexplored, it has already found applications in multiple fields. In dermatology, it is used for treating skin infections like dermatitis and ulcers. In dentistry, it serves as a natural remedy for gingivitis and periodontitis, and it is also utilized in veterinary medicine for wound care. Ozone has shown antimicrobial activity against a broad spectrum of microorganisms including bacteria, viruses, protozoa, and fungi. Additionally, it has immunomodulatory, anti-hypoxic, biosynthetic, and anti-inflammatory properties [46]. When bacteria are exposed to ozone in vitro, it oxidizes the phospholipids and lipoproteins in their cell membranes, compromising their integrity. This allows ozone to penetrate and oxidize internal components such as glycoproteins and glycolipids, thereby inhibiting crucial enzymatic functions [46]. In our study, the observed increase in yeast viability with sorbitol, but not ergosterol, suggests that ozonated olive oil primarily disrupts cell wall biosynthesis rather than the cell membrane. This alteration is significant because the cell wall is involved in both biofilm formation and the development of pseudohyphae, which are critical for the pathogenicity of *C. albicans* [47]. Electron microscopy further confirmed this, revealing substantial alterations in cell wall thickness and structure in treated cells. These modifications are likely linked to the observed reduction in biofilm formation and the suppression of pseudohyphal development, highlighting the central role of cell wall integrity in *C. albicans* virulence and the therapeutic potential of ozonated olive oil in targeting these processes. The treatment also led to elevated ROS levels, highlighting cell oxidative stress. This stress likely contributes to the antifungal action of ozonated olive oil, as ROS can damage essential cellular components, culminating in cell death. Even with the addition of ascorbic acid, a powerful antioxidant agent, ROS levels remained elevated, suggesting that ozonated olive oil triggers a potent oxidative stress. Moreover, the upregulation of autophagy-related genes and the presence of autophagosomes in treated cells suggest that ozonated olive oil may induce autophagy as an initial stress response mechanism [48]. The increased autophagy observed as early as 15 min after treatment indicates that *C. albicans* cells activate this survival mechanism to counteract the oxidative stress and damage caused by the ozonated oil. However, despite this early activation of autophagy, the cells ultimately succumb to the treatment, as evidenced by their subsequent cell death. This suggests that while autophagy is initially triggered as a protective response, it is insufficient to prevent the overall detrimental effects of ozonated olive oil, thereby contributing to its antifungal efficacy.

Despite the germicidal action, there is strong evidence supporting the biocompatibility of ozone with epithelial cells, gingival fibroblasts, and periodontal cells, indicating no risks associated with the prolonged use of ozone-based products. In fact, ozone can benefit oral tissues by promoting the remission of mucosal lesions and enhancing the turnover of oral epithelial cells, thus accelerating wound healing (45). Our results on HaCaT cells are in line with the biocompatibility of ozonated olive oil. Moreover, the cytotoxic activity of ozonated olive oil against L929 fibroblasts was assessed when the peroxide value of the compound was found to be in the range of 2700–2900 mEq O_2_/kg oil, and cell death was observed [49].

## 5. Conclusions

Overall, the results of this study indicate that ozonated EOO is a potent antifungal agent against *Candida albicans*, demonstrating efficacy in reducing cell viability, disrupting cell wall integrity, inducing oxidative stress and autophagy, and inhibiting both hyphal formation and biofilm development. These findings suggest that EOO may also be an effective antifungal agent against *C. glabrata*, potentially offering an alternative therapeutic approach for managing infections caused by non-*albicans* species that are resistant to conventional therapies. Importantly, this study identifies an optimal formulation of ozonated oil with specific concentrations and ozonide levels that maximize antimicrobial efficacy while preserving cell biocompatibility. This highlights ozonated olive oil’s potential as a promising therapeutic option, especially in the context of increasing antifungal resistance.

## Figures and Tables

**Table 1:** Triglyceride composition of ozonated extra-virgin olive oil (853 ozonides).

Compounds	Extravirgin Olive Oil	Ozonated Olive Oil 853 Ozonides (25 °C)
Nonanal	-	10.2%
Methyl ester of nonanoic acid	-	2.92%
Methyl ester of palmitic acid	12.96%	12.81%
Linoleic acid methyl ester	3.95%	1.89%
*cis*-oleic acid methyl ester	80.31%	47.42%
*trans*-oleic acid methyl ester	-	20.71%
Stearic acid methyl ester	2.78%	4.05%

## Data Availability

The data presented in this study are available upon request from the corresponding author.
